# Exploring the Activity of a Novel *N*-Glycosidase (EndoBI-2): Recombinant Production to Release Bioactive Glycans

**DOI:** 10.3390/ijms27010339

**Published:** 2025-12-28

**Authors:** Hatice Duman, İzzet Avcı, Bekir Salih, Hacı Mehmet Kayılı, Mikhael Bechelany, Sercan Karav

**Affiliations:** 1 Department of Molecular Biology and Genetics, Çanakkale Onsekiz Mart University, 17100 Canakkale, Türkiye; hatice.duman@comu.edu.tr; 2 Department of Chemistry, Faculty of Science, Hacettepe University, 06800 Ankara, Türkiye; izzetavci35@gmail.com (İ.A.); bekir@hacettepe.edu.tr (B.S.); 3 Department of Biomedical Engineering, Faculty of Natural Science and Engineering, Karabük University, 78000 Karabük, Türkiye; h.mehmetkayili@karabuk.edu.tr; 4 Institut Européen des Membranes (IEM), UMR 5635, University Montpellier, ENSCM, CNRS, F-34095 Montpellier, France; 5 Department of Medical Research, China Medical University Hospital, China Medical University, Taichung 40447, Taiwan

**Keywords:** endo-*β-N*-acetylglucosaminidase, glycan profiling, glycoprotein, deglycosylation, glycobiotechnology, mass spectrometry

## Abstract

The gut microbiome evolves in response to host development, health state, lifestyle, nutrition, and microbial interactions. The survival of gut microbiota depends on its ability to utilize its host-indigestible complex oligosaccharides. Certain gut microbes produce glycosidases that cleave *N*-glycoproteins to release *N*-glycans that are then used as a carbon source. However, commercial glycosidases are inefficient and, thus, require improved deglycosylation strategies to study their functions and scale up their production. Therefore, the main objective of this study was to recombinantly produce and characterize the novel endo-*β-N*-acetylglucosaminidase 2 (EndoBI-2) from *Bifidobacterium longum* subsp. *infantis* (*B. infantis*) and to evaluate its enzymatic performance for controlled *N*-glycan release. Furthermore, the optimum reaction conditions for EndoBI-2 were investigated on model glycoprotein RNAse B using model glycoprotein. The released *N*-glycans were profiled by hydrophilic interaction liquid chromatography-fluorescence detection-quadrupole time-of-flight tandem mass spectrometry (HILIC-FLD-QTOF-MS/MS). We demonstrated that EndoBI-2 possesses a strong temperature tolerance and efficiently cleaves *N*-glycans under mild reaction conditions, exhibiting high activity at pH 5. These findings highlight EndoBI-2 as a robust and efficient biocatalyst for the production of bioactive *N*-glycans from diverse *N*-glycoproteins, with potential applications in glycobiotechnology.

## 1. Introduction

The human gut microbiota (HGM) is a dynamic ecosystem [1,2]. The stability and functionality of HGM depend on the ability of specific microorganisms to utilize complex, host-derived glycans that are otherwise indigestible by humans. *N*-glycans attached to glycoproteins play crucial biological roles in cellular communication, immune modulation, and metabolism [3,4].

The glycosylation of mammalian milk proteins is a ubiquitous and vital post-translational modification that affects their folding, structural stability, targeting, and their final biological functions [5,6,7]. Oligosaccharides covalently linked to proteins are called glycans, which exhibit enormously diverse structures. These glycans can be connected to the protein through either *N*-glycosidic or *O*-glycosidic linkages [8]. A protein’s hydroxyl group is linked to *O*-linked glycans (*O*-glycans) by the serine (Ser) or threonine (Thr) residues via *N*-acetylgalactosamine; *N*-linked glycans (*N*-glycans) are conjugated to proteins’ asparagine (Asn) residues’ amide groups via *N*-acetylglucosamine (GlcNAc) in the Asn-X-Ser/Thr or Asn-X-Cys (cysteine) (where X can be any amino acid, with the exception of proline) [9]. Basically, the *N*-glycan core is composed of two *N*-acetylglucosamine (GlcNAc) and three mannose (Man) residues. According to their composition, additional glycosylation divides *N*-glycans into three main types including high-mannose (HM), hybrid, and complex. HM glycans typically have five to nine main residues connected to the chitobiose (GlcNAc_2_) core, along with unsubstituted terminal mannose sugars. Hybrid glycans are characterized by the partial replacement of mannose residues with GlcNAc units. GlcNAc residues are found in complex-type glycans. The diversity of glycan functions depends on the structural diversity of glycans, including the combination of constituent monosaccharides and variations in branching modes [8,10].

In glycobiology, traditional chemical methods are often used to deglycosylate glycoproteins [11]. The two most popular methods are hydrazination and β-elimination [12]. Chemical methodologies employed in glycan research possess numerous drawbacks. Alkaline conditions and reducing chemicals such as sodium borohydride can adversely affect glycans. The reducing agent converts labeling groups into alditols, complicating the attachment of fluorophores or chromophores. Monitoring the deglycosylation process is arduous, and elevated salinity might result in considerable sample losses [13]. Hydrazine treatment is a chemical deglycosylation method that enables the release of *N*-glycans from glycoproteins but requires harsh reaction conditions that may compromise protein integrity. By adjusting the reaction parameters, such as temperature, this method allows both *N*-glycans to be released under controlled conditions [13].

Enzymes such as peptidyl-*N*-glycosidases (PNGases) are incapable of cleaving glycans when *N*-acetylglucosamine is conjugated to fucose. PNGase activity necessitates severe conditions, such as denaturation, which may compromise the integrity of the liberated glycan and remaining polypeptide structures. Endoglycosidases, such as F1, F2, and F3, exhibit enhanced activity on native glycoproteins while demonstrating limited activity on multi-antennary glycans. The limited activity of endoglycosidases such as Endo F1-F3 toward multi-antennary complex *N*-glycans is mainly attributed to increased structural complexity and steric hindrance. While HM and hybrid *N*-glycans possess less branched architectures with more accessible chitobiose cores, complex *N*-glycans often contain multiple antennae, bisecting GlcNAc residues, and core fucosylation, all of which can hinder enzyme binding and catalytic efficiency [14]. EndoBI-1 from *Bifidobacterium longum subsp. infantis* (*B. infantis)* ATCC 15697, a key infant gut microbe, was demonstrated by Karav et al. (2015) to have the capacity to cleave the *N-N*′-diacetylchitobiose linkage present in the *N*-glycan core of entire classes of *N*-glycans [15]. Therefore, novel endo-*β-N*-acetylglucosaminidases with broader substrate specificity and enhanced stability are needed.

With this research, a novel enzyme, endo-*β-N*-acetylglucosaminidase 2 (EndoBI-2), was recombinantly produced and characterized. Compared to conventional chemical and enzymatic methods, this enzyme is highly active on the glycoproteins and releases different types of *N*-glycans, including HM and complex [16]. Despite the recognized importance of *N*-glycans as bioactive molecules with potential applications in nutrition and biotechnology, efficient and selective enzymatic tools for their release from native glycoproteins remain limited. This unique characteristic of EndoBI-2 offers a potentially useful method for generating *N*-glycans from glycoproteins such as lactoperoxidase (LPO) and other glycoproteins.

Based on these considerations, we hypothesized that EndoBI-2 possesses effective substrate specificity and can efficiently release types of *N*-glycans that are structurally different from both model and native glycoproteins under mild reaction conditions. Accordingly, the primary objective of this study was to evaluate the recombinant production, enzymatic activity, and deglycosylation capability of EndoBI-2, thereby addressing the need for robust enzymatic platforms for controlled *N*-glycan release.

## 2. Results

### 2.1. Recombinantly Produced Enzyme EndoBI-2 Shows Activity on Model Glycoprotein RNase B

**Figure 1:**
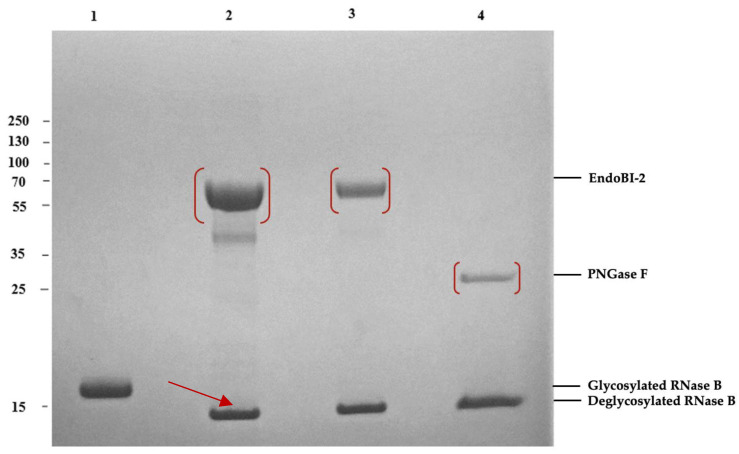
Enzymatic deglycosylation of denatured RNase B by EndoBI-2 (~58.46 kDa) and PNGase F on 4–12% SDS-PAGE gel. Lane 1: glycosylated RNase B (17 kDa). Lane 2: denatured RNase B deglycosylated by EndoBI-2 (2 µL) Lane 3: denatured RNase B deglycosylated by EndoBI-2 (0.5 µL) (14 kDa). Lane 4: denatured RNase B deglycosylated by PNGase F. Prior to enzymatic treatment, RNase B samples were denatured in Laemmli sample buffer at 95 °C for 5 min. For PNGase F reactions, denaturation was performed according to the manufacturer’s protocol, including the use of detergents.

The EndoBI-2 enzyme was successfully cloned via an *in vivo* cloning system. A comprehensive structural study of EndoBI-2 was conducted to determine its conserved domains and functional regions in our previous *in silico* study. To enhance the solubility and functional expression of the recombinant enzyme in a heterologous host, the transmembrane and signal peptide regions were removed during cloning (Figure 1) [17].

Using the model glycoprotein Ribonuclease B (RNase B), the activity of recombinant EndoBI-2 was demonstrated. RNase B, a 17 kDa protein subject to *N*-glycosylation, undergoes deglycosylation to yield a 14–15 kDa protein (Figure 1).

### 2.2. Optimizing EndoBI-2 Enzymatic Activity

**Figure 2:**
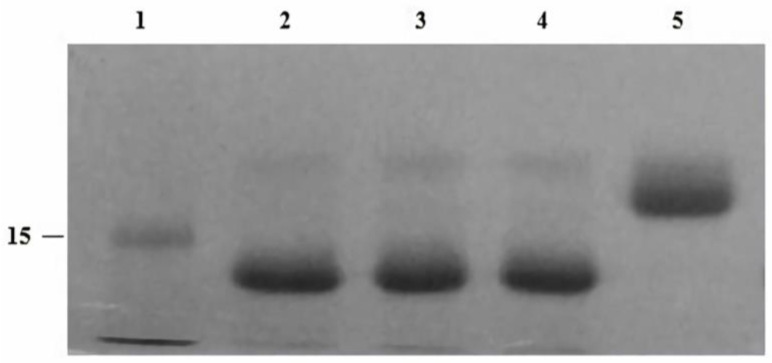
SDS-PAGE (4–12%) gel of recombinant EndoBI-2 enzyme optimization experiment. Lane 1: ladder (kDa). Lane 2: denatured RNase B deglycosylated by EndoBI-2 at 37 °C pH 5. Lane 3: denatured RNase B deglycosylated by EndoBI-2 at 30 °C pH 5. Lane 4: denatured RNase B deglycosylated by EndoBI-2 at 24 °C pH 5. Lane 5: RNase B. For all lanes, 1 µL of EndoBI-2 and RNase B (17 kDa) were loaded.

**Figure 3:**
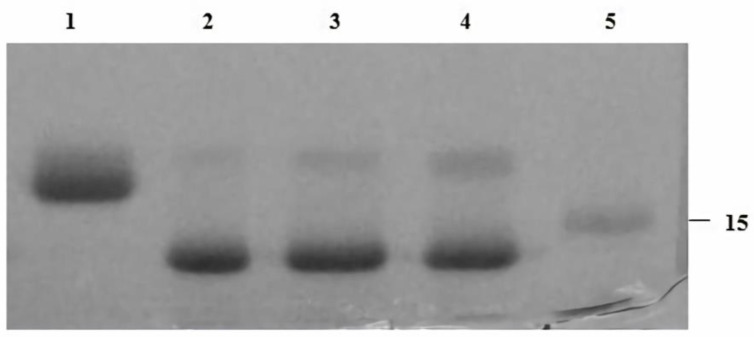
SDS-PAGE (4–12%) gel of recombinant EndoBI-2 enzyme optimization experiment. Lane 1: RNase B. Lane 2: denatured RNase B deglycosylated by EndoBI-2 at 37 °C pH 5. Lane 3: denatured RNase B deglycosylated by EndoBI-2 at 37 °C pH 6. Lane 3: denatured RNase B deglycosylated by EndoBI-2 at 37 °C pH 7. Lane 5: ladder (kDa). For all lanes, 1 µL of EndoBI-2 and RNase B (17 kDa) were loaded.

EndoBI-2 operates effectively across a wide range of temperatures (24–37 °C). The enzyme exhibited the highest activity at 37 °C and remained stable at 24 and 30 °C, indicating good thermal tolerance. However, pH changes exerted only a minor effect on EndoBI-2 activity, and it maintained an optimal performance at pH 5. Figure 2 shows that EndoBI-2 maintains its activity at pH 5 across three tested temperatures. As visible in Figure 3, when the temperature was kept at 37 °C, the enzyme acted on RNase B at pH 5, pH 6, and pH 7, maintaining high activity even at pH 5.

### 2.3. Profiling of N-Glycans Released from RNase B and LPO by EndoBI-2 Deglycosylase

**Figure 4:**
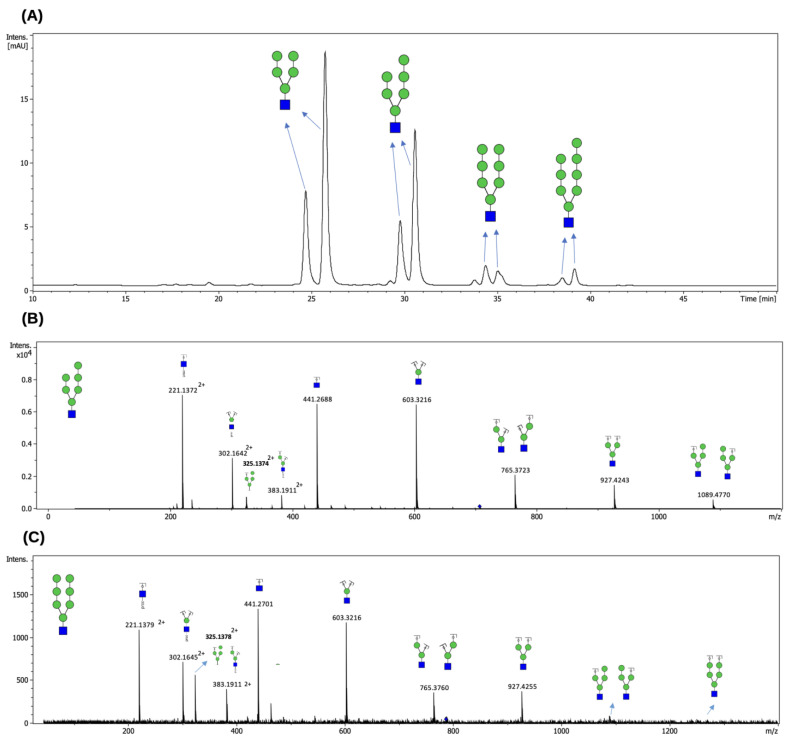
FLD chromatogram of *N*-glycans released from RNase B by EndoBI-2 (**A**), together with the corresponding MS/MS spectra of the procainamide-labeled H6N1 (**B**) and H7N1 (**C**) glycan structures. The peaks were annotated based on theoretical compositions and matched to HM glycan structures using the GlycoWorkBench v2 software. Green circles represent Man residues, and blue squares denote GlcNAc residues. The HILIC profile shows HM-type *N*-glycans released from RNase B by EndoBI-2. The identified peaks correspond to H5N1 (Man_5_GlcNAc_1_), H6N1 (Man_6_GlcNAc), H7N1 (Man_7_GlcNAc), and H8N1 (Man_8_GlcNAc). The elution order of these glycans is governed by their hydrophilicity; glycans with higher mannose branches are more polar and therefore exhibit stronger interactions with the HILIC stationary phase, leading to longer retention times. The obtained results from the analysis confirm that EndoBI-2 efficiently releases a range of HM *N*-glycans from RNase B under mild reaction conditions. EndoBI-2 exhibits a defined substrate specificity, cleaving within the core chitobiose unit of *N*-glycans and releasing structures while leaving a single GlcNAc residue attached to the asparagine, which fundamentally differentiates its action from PNGase F.

Following the optimization of enzymatic reaction parameters including pH and temperature, the deglycosylation capacity of EndoBI-2 was evaluated using RNase B as a widely recognized model *N*-glycoprotein and LPO as a native *N*-glycosylated protein. Knowing that RNase B carries exclusively HM-type *N*-glycans, we were interested in examining whether EndoBI-2 can release complex-type, terminally sialylated *N*-glycans attached to LPO. Figure 4 depicts the FLD chromatogram of HM-type *N*-glycans cleaved from RNase B (Figure 4A), along with their mass spectra (Figure 4B,C).

**Figure 5:**
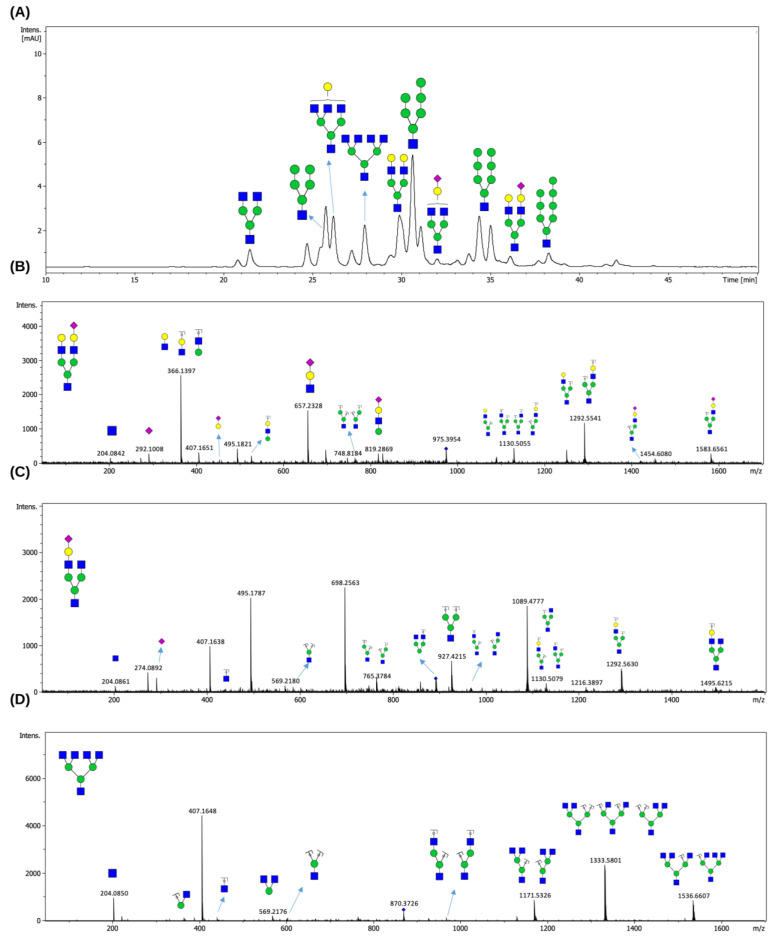
HILIC-FLD chromatogram of *N*-glycans released from LPO by EndoBI-2 (**A**). MS/MS spectra of *N*-glycans released from LPO by EndoBI-2. Panels (**B**–**D**) present representative MS/MS spectra of major complex-type *N*-glycans identified in LPO. The detected structures correspond to H5N3S1 (Man_3_GlcNAc_3_Gal_2_NeuAc), H4N3S1 (Man_3_GlcNAc_3_GalNeuAc), and H3N5 (Man_3_GlcNAc_5_). Peak annotation and structural assignment were performed using the GlycoWorkBench software v2. Green circles, yellow circles, blue squares, and purple diamonds represent Man, Gal, GlcNAc, and NeuAc residues, respectively.

Following enzymatic treatment with EndoBI-2, which specifically cleaves *N*-glycans while preserving the reducing end, the released glycans were analyzed by LC-MS/MS. Figure 5 illustrates the *N*-glycan profiling results obtained for the LPO glycoprotein. We revealed that LPO harbors a heterogeneous population of *N*-glycans, encompassing HM- and complex-type *N*-glycans.

As shown in Figure 5A, the compositions of the detected *N*-glycans were confirmed based on their accurate masses and their characteristic retention times on the HILIC column. The elution order reflects increasing hydrophilicity, which overlaps with increasing molecular mass, with sialylated *N*-glycans showing longer retention times. The MS/MS spectra presented in Figure 5B–D provided validation of the proposed *N*-glycan structures, displaying diagnostic fragment ions arising from major cleavages of glycosidic bonds, alongside cross-ring fragmentations. These fragmentation patterns enabled precise differentiation of structural features such as branching and sialylation.

## 3. Discussion

### 3.1. Biological Relevance of EndoBI-2 in N-Glycan Utilization

The major purpose of this research was to analyze the enzymatic performance of EndoBI-2 and to examine its potential to release structurally varied *N*-glycans from both model and natural glycoproteins under mild reaction conditions. In line with the experimental results obtained in this study, the discussion below focuses on placing these findings within a relevant biological and functional context.

Breastfeeding affects the composition of the newborn gut microbiota during the initial months of life, as substantial quantities of human milk oligosaccharides (HMOs) act as carbon sources for diverse bacteria such as *Bifidobacterium* species, which have evolved molecular mechanisms to metabolize these oligosaccharides [18]. Bacterial endo-*β-N*-acetylglucosaminidases (EC 3.2.1.96) play a central role in the release of *N*-glycans from glycoproteins, enabling certain gut microorganisms to utilize these complex carbohydrates as carbon sources [19,20]. Members of this enzyme group have been identified in *B*. *infantis*, *B. longum*, and *B. breve*, which are key inhabitants of the infant gut microbiota that contribute to the digestion of milk-derived glycoconjugates [8,16].

The enzymatic activity observed for EndoBI-2 in Section 2 is consistent with the biological role of endo-*β-N*-acetylglucosaminidases in facilitating access to *N*-glycans from glycoprotein substrates, thereby supporting glycan utilization pathways in glycan-adapted microorganisms.

### 3.2. Substrate Specificity and Catalytic Properties of EndoBI-2

Among these enzymes, EndoBI-2 represents a distinct member of the GH18 family with unique structural and functional properties. Unlike previously characterized endoglycosidase, EndoBI-2 exhibits broad substrate specificity and operates efficiently under mild, acidic conditions without requiring denaturation. Because of this characteristic, EndoBI-2 is distinguished from traditional deglycosylation enzymes, including PNGase F, which normally need severe reaction conditions and have limited activity toward native glycoproteins. EndoBI-2, on the other hand, possesses this property. These characteristics make EndoBI-2 a valuable biocatalyst for the selective release of bioactive *N*-glycans from native glycoproteins [16,17,21].

As demonstrated by the deglycosylation results presented in this study, the ability of EndoBI-2 to act efficiently on native glycoproteins supports its broad substrate specificity and distinguishes it functionally from conventional deglycosylation enzymes. This functional behavior provides a mechanistic basis for the observed release of structurally diverse *N*-glycans under mild reaction conditions.

### 3.3. Deglycosylation Performance on Model and Native Glycoproteins

In the present study, a novel enzyme, EndoBI-2, was recombinantly produced and characterized. Compared to conventional chemical and enzymatic methods, this enzyme is highly active on the glycoproteins and releases different types of *N*-glycans, including HM and complex structures [16]. In addition to confirming that the enzyme’s catalytic effectiveness is maintained across structurally diverse glycoproteins, the successful deglycosylation of both RNase B and LPO demonstrates the enzyme’s broad substrate tolerance. This unique catalytic capability highlights its potential as an effective biocatalyst for generating bioactive *N*-glycans from diverse glycoproteins such as LPO and lactoferrin.

The deglycosylation results obtained for RNase B and LPO, as presented in Section 2, provide direct experimental evidence supporting the broad substrate tolerance of EndoBI-2 under the applied reaction conditions. In addition, the enzymatic parameters and reaction conditions of EndoBI-2 were optimized, and the released glycans were profiled using HILIC-FLD-QTOF-MS/MS, allowing for detailed structural confirmation and comparison with previously reported *N*-glycan profiles. This analytical approach enabled a systematic evaluation of the glycan structures released by EndoBI-2 and facilitated direct comparison with established *N*-glycan datasets, thereby strengthening the interpretation of the experimental findings.

The HILIC profile in Figure 4A demonstrates that EndoBI-2 effectively cleaves HM *N*-glycans from RNase B. In our HILIC data, the same HM precursor appeared at multiple, closely spaced retention times, indicating chromatographically distinct species. Although these forms displayed nearly identical MS/MS fragmentation patterns, their differing HILIC retention supports the presence of structural isomers. Our results are consistent with the *N*-glycan profile reported by Prien et al. (2008) [22], who identified Man_5_GlcNAc_2_, Man_7_GlcNAc_2_, and Man_8_GlcNAc_2_ as the major *N-*glycans of RNase B. As expected, the use of EndoBI-2 in our study resulted in the release of Man_5_-Man_8_GlcNAc structures (rather than Man_5_-Man_8_GlcNAc_2_), due to cleavage between the two core GlcNAc residues. Moreover, the HILIC and MS/MS data revealed multiple isomeric forms of HM glycans, which further supports the structural heterogeneity previously described for RNase [22].

On the other hand, our findings suggest that EndoBI-2 efficiently releases both asialo and sialylated *N*-glycans from native LPO under mild reaction conditions, thus confirming its relatively broad substrate specificity (Figure 5). Overall, the combined use of EndoBI-2 digestion and LC-MS/MS enabled reliable detection and compositional characterization of the *N*-glycans released from LPO. These observations are consistent with the detailed *N*-glycan characterization reported by Wolf et al. (2000) [23], who demonstrated that bovine LPO carries predominantly HM and complex-type glycans, several of which were also detected in our analysis. Although Wolf et al. reported core-fucosylated structures, such species were not observed here because EndoBI-2 cleaves within the core chitobiose and removes the innermost GlcNAc–Fuc moiety, thereby preventing the detection of core fucose in the released glycan pool [17]. This characteristic, however, may represent an analytical advantage in applications where selective cleavage of core-fucosylated *N*-glycans is desirable while preserving antennary fucosylation for downstream structural or functional studies.

### 3.4. Structural Features Contributing to Enzymatic Functionality

Structural analysis of this enzyme revealed that its modular architecture enhances its potential as a selective glycan-binding biocatalyst. The carbohydrate-binding modules (CBMs) within EndoBI-2 can be interchanged with those from other GH18 family members or modified at the + subsite to improve substrate-binding affinity. Furthermore, catalytically inactive variants can be engineered to facilitate purification and functional characterization. The extensive substrate specificity that was seen experimentally can be explained by a mechanistic explanation that is provided by such modularity.

This modular architecture aligns with the functional diversity of EndoBI-2 and offers a structural foundation that facilitates varied substrate identification within the GH18 enzyme family. Nevertheless, neither model was able to capture the β-sandwich framework of the CBM 32 domains, indicating that the thermostability of these enzymes may be affected by their distinct CBM structure. The architecture of its CBM region may contribute to its broad substrate specificity and thermostability, which are characteristics that enhance its functionality in diverse reaction environments. These features position EndoBI-2 as a promising and food-grade enzyme candidate for applications in glycobiology and industrial glycan production [17].

These structural considerations frame the enzymatic action of EndoBI-2 found in our investigation, without suggesting further experimental structural characterization beyond previously published findings.

### 3.5. Influence of Reaction Parameters on EndoBI-2 Activity

The EndoBI-2 enzyme was isolated from the gut microbiome of *B. infantis* utilizing an *in vivo* cloning technique. Structural analysis revealed conserved catalytic domains, which facilitated efficient recombinant expression of the enzyme in our study [17]. Optimization experiments demonstrated that temperature strongly influenced the deglycosylation efficiency of EndoBI-2 toward RNase B. The enzyme retained high activity across a broad temperature range, while pH variation had minimal effect on its performance. EndoBI-2 demonstrates a degree of functional resilience that is desired for regulated enzymatic applications, as evidenced by its capacity to maintain enzymatic activity in conditions that are moderately acidic and throughout a wide temperature range.

EndoBI-2’s relevance in industrial and biotechnological processes that favor moderate and regulated reaction conditions is further supported by the fact that it has been discovered to have a specificity for pH 5 and to maintain activity throughout a wide temperature range. Such stability under mild reaction conditions may facilitate its use in analytical and preparative settings where harsh conditions are undesirable.

The present study focused on the *in vitro* enzymatic characterization of EndoBI-2 using selected model and native glycoproteins. It is recommended that future studies concentrate on assessing the efficacy of EndoBI-2 with regard to a wider variety of glycoprotein substrates, as well as investigate its scalability and incorporate it into preparative processes. In addition, evaluating the functional and biological features of the *N*-glycans that have been released in the appropriate biological models would give more insight into the prospective uses of these molecules. Such studies will help to better define the scope and applicability of EndoBI-2 in glycobiotechnology and functional food research.

## 4. Materials and Methods

This present study was designed as an *in vitro* experimental enzymatic study to evaluate the activity, substrate specificity, and deglycosylation efficiency of the recombinant EndoBI-2 enzyme. The experimental workflow included recombinant enzyme production, enzymatic deglycosylation assays using model and native glycoproteins, optimization of reaction parameters, and structural analysis of released *N*-glycans.

### 4.1. Reagents, Enzymes, and Substrates

The enzymatic deglycosylation experiments were conducted using LPO from bovine milk (Sigma-Aldrich, St. Louis, MO, USA.). RNase B and PNGase F (New England Biolabs, Ipswich, MA, USA) were employed as a model glycoprotein and a reference enzyme, respectively, to verify enzymatic activity. RNase B was selected as a model glycoprotein containing exclusively HM-type *N*-glycans, whereas LPO was used as a native glycoprotein carrying complex and sialylated *N*-glycans. The Qubit™ Protein Assay Kit and HisPur™ Ni-NTA Resin were obtained from Thermo Fisher Scientific (Waltham, MA, USA) [15].

### 4.2. Gene Cloning, Expression, and Purification

The gene cloning process was performed using an Expresso^®^ Rhamnose Cloning and Protein Expression *in vivo* system (Lucigen Corp., Middleton, WI, USA), with adherence to the manufacturer’s protocols [24]. The coding sequence of EndoBI-2 (BLIF_1310; EC 3.2.1.96) was amplified from *B. infantis* 157F using appropriate cloning primers *(*5′- CGCGAACAGATTGGAGGTGTTGCGAACGCCCAGGAG-3′ and 5′-GTGGCGGCCGCTCTATTACGCCGCGTTTCTGGCCGT-3′) and then cloned into the pRham™ N-His SUMO Kan vector. The enzyme was cloned by adding an N-terminal poly-histidine SUMO tag without transmembrane domains and signal peptide sequences to facilitate protein expression and purification. Poly-histidine-SUMO-tagged EndoBI-2 was produced in the *E. coli* host on LB medium under optimal induction conditions (0.2% final concentration L-rhamnose, 37 °C for 18–24 h). The sequence was confirmed using sequencing primers (SUMO forward; 5′-ATTCAAGCTGATCAGACCCCTGAA-3′, pETite^®^-reverse; 5′-TCAAGACCCGTTTAGAGGC-3). His-tagged protein was purified on nickel-charged affinity column and eluted by 250 mM imidazole solution. Enzyme was visualized on SDS-PAGE gradient gels (4–12%) and its concentration was determined by a Qubit™ Protein Assay Kit.

### 4.3. EndoBI-2 Enzymatic Activity Using Model Glycoprotein

To compare the enzymatic activities of EndoBI-2 and a convenient *N*-glycosidase PNGase F, we employed the common substrate, denatured RNase B. A total of 5 µg of RNase B was denatured by incubation at 95 °C for 5 min. The denatured RNase B samples were incubated for 1.5 h at 37 °C in a shaking incubator (200 rpm) with EndoBI-2, which was added at a final enzyme concentration of 0.025 mg/mL, in 0.02 M Na_2_HPO_4_ buffer (pH 5). In the present assay, PNGase F was used as a positive control. For PNGase F reaction, RNase B was denatured in a glycoprotein denaturing buffer (10× concentrated stock solution) at 95 °C for 10 min. The denatured RNase B sample underwent incubation at 37 °C for 1.5 h (h) with PNGase F in Glycobuffer (10×) and a 10% NP-40 solution. Protein denaturation prior to PNGase F treatment ensures full accessibility of *N*-glycosylation sites, while detergent-based denaturation does not alter *N*-glycan structures. Following the incubation period, samples from the enzyme treatment were examined on 4–12% Bis-Tris precast SDS-PAGE gels (Invitrogen, CA, USA) and stained for 30 min using Coomassie brilliant blue solution [25].

### 4.4. Enzyme Optimization Studies of EndoBI-2

To optimize the enzymatic reaction conditions of EndoBI-2 on the RNase B (5 mg/mL), various pH/temperature conditions were investigated and combined. RNase B was used as the model substrate for all optimization experiments. Enzymatic reactions employing EndoBI-2 were conducted under constant pH (pH 5) with varying temperatures (24 °C, 30 °C, and 37 °C), as well as under constant temperature (37 °C) with varying pH levels (pH 5, 6, and 7). The reactions were allowed to proceed for 1 h prior to termination [15].

### 4.5. N-Glycans Profiling Using Hydrophilic Interaction Liquid Chromatography–Fluorescence Detection–Quadrupole Time-of-Flight Tandem Mass Spectrometry (HILIC-FLD-QTOF-MS/MS)

#### 4.5.1. Deglycosylaton of Glycoproteins

The first 5 mg/mL of LPO was deglycosylated using EndoBI-2 (0.025 mg/mL). The enzyme was incubated in 0.02 M Na_2_HPO_4_ buffer solution under optimized conditions (37 °C, pH 5, and 1 h). After the reaction was terminated and the released glycans were collected by protein precipitation using cold ethanol into the sample (4:1 *v*/*v*), following the manufacturer’s instructions, the Qubit protein assay kit was used to determine the concentrations of the substrate and enzyme [25].

#### 4.5.2. Procainamide Labeling

The procainamide labeling of *N*-glycans was performed with slight modifications based on previously described methods [26]. Briefly, samples (25 μL) were mixed with 12.5 μL of procainamide hydrochloride (38.3 mg/mL in DMSO/acetic acid, 7:3, *v*/*v*) and 12.5 μL of picoline borane (44.8 mg/mL in DMSO/acetic acid, 7:3, *v*/*v*). The reaction mixtures were then incubated at 65 °C for 2 h.

#### 4.5.3. Purification of Procainamide-Labeled *N*-Glycans

A small piece of cotton wool was inserted into the tip of a 10–100 μL pipette. The cotton-containing pipette tip was pre-washed by five sequential rinses with 100% water followed by 85% acetonitrile (ACN). Subsequently, 30 μL of procainamide-labeled *N-*glycan solution was mixed with 170 μL of ACN to prepare the loading solution. Each sample was loaded by repeatedly aspirating and dispensing (20–25 cycles) through the cotton-packed pipette tip. The tips were then washed five times with 100 μL of 85/14/1 (*v*/*v*/*v*) ACN/water/trifluoroacetic acid (TFA) and 85/15 (*v*/*v*) ACN/water solutions, respectively. Finally, the retained *N*-glycans were eluted by pipetting up and down 20–25 times with 25 μL of 100% water. The purified samples were subsequently prepared for analysis.

#### 4.5.4. HPLC-HILIC-FLD-QTOF-MS/MS Analysis of *N*-Glycans

The procainamide-labeled *N*-glycans were analyzed using hydrophilic interaction liquid chromatography (HILIC) on an Agilent 1200 Series high-performance liquid chromatography system equipped with a fluorescence detector, which was interfaced with a quadrupole time-of-flight mass spectrometer (timsTOF, Bruker Daltonics, GmbH).

The labeled *N*-glycans were separated on the Waters Glycan BEH Amide column (1.7 µm, 2.1 mm × 150 mm). The mobile phases consisted of 100% ACN (solvent A) and 50 mM ammonium formate (pH 4.4) (solvent B). The gradient for solvent A was linearly decreased from 75% to 25% over 65 min at a constant flow rate of 0.25 mL/min. A total volume of 40 μL was injected for each analysis. The excitation and emission wavelengths of the FLD detector were set to 310 nm and 370 nm, respectively.

The LC-MS system was controlled using the HyStar 4.1 software (Bruker Daltonics, GmbH). Mass spectrometric detection was performed on a timsTOF instrument operated strictly in QTOF mode, without activating the TIMS ion mobility module. The ion source parameters were set as follows: capillary voltage 4.5 kV, source temperature 250 °C, nebulizer gas pressure 1.8 bar, and drying gas flow 7 L/min. MS data were acquired over an *m*/*z* range of 50–2800 with a scan rate of 0.5 Hz, providing sufficient spectral density for glycan compositional analysis. The preferred mass range (500–2500 *m*/*z*) was used for precursor selection. Singly charged ions and ions with unknown charge states were excluded, while precursors with charge states between +2 and +5 were preferentially isolated. A group length of five spectra was applied for precursor targeting. Active exclusion was enabled to prevent repeated fragmentation of the same precursor and to maximize glycan coverage.

Profiling of the identified *N*-glycans was carried out based on their MS spectral features using the GlycoWorkBench software. The reference glycan library included structures composed of the common *N*-glycan monosaccharides (Man, Gal, GlcNAc, Fuc, NeuAc and NeuGc) [26].

## 5. Conclusions

Deglycosylation represents an important analytical and biotechnological approach for the release of *N*-glycans from glycoproteins. In this study, the novel enzyme EndoBI-2 was recombinantly produced and characterized as an *N*-glycosidase capable of releasing *N*-glycans from glycoprotein substrates.

The experimental strategy comprised recombinant enzyme production, optimization of reaction parameters, and systematic profiling of released *N*-glycans. EndoBI-2 demonstrated effective deglycosylation activity under mild reaction conditions, resulting in the release of structurally diverse *N*-glycans.

Overall, this work establishes EndoBI-2 as a reliable enzymatic tool for controlled *N*-glycan release and provides a foundation for further investigations into its application in glycobiotechnology and functional food research.

## Data Availability

The original contributions presented in this study are included in the article. Further inquiries can be directed to the corresponding authors.
